# The Twenty-Seventh Long Fox Memorial Lecture: Air as a Conductor of Electricity

**Published:** 1938

**Authors:** A. M. Tyndall

**Affiliations:** Henry Overton Wills Professor of Physics in the University of Bristol


### The Bristol Medico-Chirurgical Journal

*"Scire est nescire, nisi id me*
*Scire alius sciret."*

WINTER, 1938.

### THE TWENTY-SEVENTH LONG FOX MEMORIAL LECTURE:

DELIVERED IN THE UNIVERSITY OF BRISTOL ON TUESDAY, 15th NOVEMBER, 1938.

BY

PROFESSOR A. M. TYNDALL, D.Sc., F.R.S.,
*Henry Overton Wills Professor of Physics in the University of Bristol.*

ON

### AIR AS A CONDUCTOR OF ELECTRICITY.

SINCE an electrified body normally retains its charge when surrounded by air, the air in its ordinary state must be an exceedingly good insulator. But it may be made conducting in a variety of ways. Thus it may easily be demonstrated* that the leaves of an electroscope gradually fall when a lighted match is held near to it or when the radiations from radium

* The use of the word "demonstration" in this account covers some of the experiments shewn in the lecture.

or an X-ray tube pass through the air surrounding it. The penetrating power of X-rays can be shown in this way across a large lecture theatre.

The conducting properties of the air may be explained in the following way: On modern views an atom consists of a central positively charged nucleus where its mass is concentrated, surrounded by a number of negative electrons. The two charges are equal, so that the atom, treated as a whole, is electrically neutral. The charge on the nucleus determines the element. Thus hydrogen has 1 positive charge, helium 2, lithium 3, and uranium, the heaviest element, 92. When a train of short electromagnetic waves such as X-rays or the gamma rays from radium is absorbed by an atom, the result is that one of its electrons is shot out at high speed. We thus produce in the gas two carriers of electricity, one a negative ion (electron), and the other the original atom bereft of one negative charge and therefore a positive ion. The high speed electron, however, can also produce a similar though less violent disturbance in atoms near which, or even through which, it passes. It may travel in this way a considerable distance before it comes to rest and will leave in its path a number of pairs of ions which define its trail. By making use of the property of ions that they can act as nuclei for the condensation of mist, these particles can be loaded up to a visible size. All that is necessary to see these tracks is to carry out the experiment in moist gas and then suddenly cool it by a rapid expansion.

Other ionizing projectiles may be used, such as hydrogen nuclei (protons), which are hydrogen atoms from which the electron has been removed. Alpha particles, which were first discovered by their emission from radium in its natural disintegration, show very pronounced tracks because of their greater mass and ionizing power ; they are helium nuclei.

The production in this manner of a number of ions in air is all that is necessary to give it conducting properties, because they carry charges and can thus give rise to an electric current between two electrodes to which a voltage is applied. The movement of these ions can be copied as a demonstration on a magnified scale by using coloured powders of electrified red lead and yellow sulphur and also by electrified soap bubbles.

When the voltage is greatly raised, then any ions which may be present acquire a high speed and act themselves as ionizing projectiles. The new ions so produced repeat the process and in this way it rapidly works up to explosive violence giving rise to a spark. The voltage necessary to give rise to a spark is reduced if a number of ions are supplied to the spark gap from another source to start off the process. This effect may easily be demonstrated and is the basis of the action of the "clucker" installed at the Bristol General Hospital as a detector of radium. In this instrument an electrical circuit is arranged so that a succession of flashes of glow occur in a neon lamp, each giving a "cluck" in a loud speaker. If the voltage is reduced so that these flashes just fail to pass, and if radium is then brought near to the lamp, the gamma rays from the radium produce ions in the neon gas in the lamp, so that the applied voltage is now sufficient for the succession of discharges to take place. The clucker may thus be used rapidly to test dressings, or refuse from an incinerator, for radium suspected to be missing.

These processes of ionizing a gas do not in general result in the "splitting of an atom." To alter an atom fundamentally one must alter its central nucleus, and to do this one must make a direct hit. This is now being done using intense beams of high speed protons and also neutrons, a valuable new particle, which has a nucleus of the same mass as hydrogen, but no electric charge.

In many cases it is found that a projectile making a direct hit may enter the nucleus of the atom that it strikes, thus adding to its mass and its charge. The result is expressed in a new type of chemical equation in which we have to equate the masses as well as the charges of the colliding nuclei on the two sides of the equation. The resultant nucleus is sometimes so unstable that it immediately disintegrates, giving out a high speed particle which may be recognized by the length and density of its ionization track. An example of this, historically the first, was obtained by bombarding nitrogen with alpha particles—

$$^{14}_{7}\mathrm{N} + ^{4}_{2}\mathrm{He} \longrightarrow ^{17}_{8}\mathrm{O} + ^{1}_{1}\mathrm{H}$$

The figures on the top left represent the masses of the atoms. When the alpha particle enters the nucleus of nitrogen an atom of mass 18 would be produced. But this is completely unstable and ejects a proton of mass unity leaving one of the isotopes of oxygen of mass 17. The bottom left hand figures give the charges involved and it will be seen that these also balance.

In some cases the resultant new atom does not immediately break up, but behaves like radium in that it may break up at any moment. Thus, if large numbers are produced, some will be breaking up all the time over an extended period, the rate of breaking up falling off as the number of *unbroken* atoms decreases. This is the phenomenon of artificial radioactivity, examples of which are now very numerous.

A typical example is obtained by firing neutrons at a sodium salt; this gives rise to a new variety of sodium possessing the same chemical properties as ordinary sodium but having atoms of mass 24 instead of 23. The equation is as follows:—

$$^{23}_{11}\text{Na} + {}^{1}_{0}\text{neutron} \longrightarrow {}^{24}_{11}\text{Na}$$

Sodium 24 is not permanently stable and sooner or later changes into non-radioactive magnesium with the emission of a high speed electron or beta ray.

$$^{24}_{11}\text{Na} \longrightarrow {}^{24}_{12}\text{Mg} + {}^{0}_{-1}\text{electron}$$

The rate of decay of radio-active sodium is such that half of it disappears in $14\frac{1}{2}$ hours, three-quarters of it in 29 hours, and seven-eighths of it in 58 hours. Since the "half-life" for radium is 1,700 years, it is possible to make a quantity of sodium which is enormously more powerful than the same mass of radium, but only, of course, for a limited period. In this way samples of radio-active common salt have been artificially made in one experiment equal in power to nearly half a gram of radium. It is possible not only to demonstrate the emission of the high-speed particles during the disintegration, but also actually to count the number emitted in a given time.

Another element likely to be of great biological use is radio-active phosphorus, which has a "half-life" of $14\frac{1}{2}$ days. It is therefore not so powerful as sodium, but its longer life increases its applicability.

These new radio-active elements are now being applied to biological problems, because they produce a method of tracing biological processes by atoms which are labelled by their radio-active properties. Indeed, they show promise of throwing much light on many outstanding problems in metabolism. Thus, if some radio-active phosphate is added to a phosphate meal given to an animal, the time that elapses before the phosphorus atoms enter the bones, the teeth, or other organism, can be determined. For instance, it has already been shown that fully synthesized radio-active lecithin is found in the brain tissue of a fully-grown rat only one hour after a sodium phosphate injection has been administered. Information of this type cannot be acquired by any other known method. Similarly the percentage of atoms eaten which subsequently enter any given structure may be estimated. Experiments also on the effect on these quantities of diet or disease are brought within the range of attack.

The possibility of the use of radio-active elements as substitutes for radium in radium therapy must be regarded at present as an open question. But some radio-active elements emit intense gamma rays. If such an element could be produced *in situ* in any affected part of the body by injecting the appropriate parent element and then bombarding it with neutrons, it might be a most effective agent.

It is clear that we are witnessing the birth of an entirely new development of profound significance. It may well prove to be one of the most important contributions ever made by physics to medical research.

